# Attitude, Literacy, and Social Stigma Toward Mental Illness Among Islamic Religious Leaders (Imams) in Bangladesh: A Cross‐Sectional Study

**DOI:** 10.1002/brb3.70998

**Published:** 2025-10-15

**Authors:** S. M. Yasir Arafat, Md. Khayrul Islam, Sanjida Tanjin Khan, Md. Golam Kibria, Bithika Mali

**Affiliations:** ^1^ Department of Psychiatry Bangladesh Specialized Hospital Dhaka Bangladesh; ^2^ Department of Public and Community Health Faculty of Medicine and Health Sciences Frontier University Garowe Garowe Somalia; ^3^ Department of Psychiatry Tairunnessa Memorial Medical College Gazipur Bangladesh; ^4^ Department of Psychiatry Ad‐din Medical College and Hospital Dhaka Bangladesh; ^5^ Department of Research Centre For Development Action Dhaka Bangladeshs; ^6^ Department of Graduate Nursing Faculty of Nursing Bangladesh Medical University Dhaka Bangladesh

**Keywords:** attitude, Bangladesh, Imam, literacy, mental illness, religious leaders, stigma

## Abstract

**Background:**

More than 90% of people in Bangladesh are Muslims and a good portion of persons with mental illness visit religious leaders in the country. Attitude, literacy, and stigma toward mental illness affect the referral of persons with mental illness to mental health systems.

**Objectives:**

We aimed to see attitudes, literacy, and social stigma toward mental illness among Islamic religious leaders in Bangladesh.

**Methods:**

Data were collected for this cross‐sectional study by a self‐reporting semi‐structured questionnaire from 358 Imams by convenience sampling from September 2024 to January 2025.

**Results:**

The mean age of the participants was 31.7 years. The majority of the participants were aged 30 years or younger (54.5%), were married (68.4%), maulanas (59.8%), and 8.1% had a history of psychiatric illness. Mental illness was perceived as highly biological, influenced by sinful acts and spiritual influence, and was poorly associated with external issues like jinn, evil eye, and black magic by the participants. A favorable scenario was noted while assessing the social stigma of mental illness, while persons with mental illness were considered dangerous. Participants correctly identified depression (92.5%), anxiety (90%), obsessive–compulsive disorder (83%), and Schizophrenia (88%).

**Conclusions:**

This study revealed findings related to attitude, social stigma, and literacy among religious leaders in a Muslim‐majority country. Studies assessing the association between these variables and referral patterns to mental health services are warranted to consider the findings in policymaking.

## Graphical abstract

This paper assessed the attitudes, literacy, and social stigma towards mental illness among Islamic religious leaders in Bangladesh. Mental illness was perceived as highly biological, influenced by sinful acts and spiritual influence, and was poorly associated with external issues like jinn, evil eye, and black magic by the participants. A favorable scenario was noted while assessing the social stigma of mental illness, while persons with mental illness were considered as dangerous.

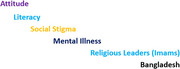

## 1 Introduction

Religious leaders play significant roles in the society. People share their distress, personal hidden thoughts, and sufferings with religious leaders (Ali and Milstein 2012). Islamic religious leaders (Imams) can influence beliefs, attitudes, and behavior of Muslims living in the community (Suleiman et al. 2023; Long and Ansari 2018; Sabry and Vohra 2013). Thus, Imams play a vital role by influencing and suggesting the treatment methods and modes for mental illness in the Muslim community (Meran and Mason 2019).

Bangladesh is a lower‐middle‐income country in South Asia with about 170 million people (World Bank 2024; Bangladesh Bureau of Statistics 2024). More than 90% of its population is Muslim, and about 70% live in rural areas (Bangladesh Bureau of Statistics 2024). The prevalence of psychiatric disorders is 18.7% where there is a treatment gap of more than 90% (Ministry of Health and Family Welfare 2021). Studies reported that about one‐fourth of psychiatric patients visited religious leaders/traditional healers as their first contact for services (Giasuddin et al. 2012; M. M. Islam et al. 2001). Additionally, as pertinent in the religion (Islam), Jinn is another creature of Allah, and psychiatric symptoms have been attributed to Jinn possession in Bangladesh (Selim and Satalkar 2008; Mullick et al. 2013). The findings indicate the role of religious leaders in mental health care in Bangladesh.

Mental health literacy (MHL) is an important aspect affecting care‐seeking, referral to mental health care, and personal stigma (Elyamani and Hammoud 2020; Krstanoska‐Blazeska et al. 2021; Guden et al. 2024). Literacy shapes personal stigma, stigma toward persons with mental illness, and attitude toward persons with mental illness. There are several aspects of mental illness that affect care‐seeking behavior among Muslims, such as mental illness can be caused by the divine will or from Allah as a test or punishment, and can be caused by Jinn, evil eye, and black magic (Hussain 2022). Imams as religious leaders can treat mental illness by prayer (Dua) and therefore, logically, people seek help from religious leaders in Bangladesh for mental symptoms. Positive attitude, absence of social stigma, and improved literacy of mental illness play beneficial roles by referring persons with mental symptoms to available mental health services. Adequate knowledge about mental illness and its treatment shapes the attitude and reduces the stigma as mental symptoms are attributed to supernatural power, black magic, and evil eye, where Imams play an active role in addressing the supernatural power, controlling the evil eyes, and black magic.

Bangladesh is the fourth leading country in terms of the number of Muslims and mosques in the world. It has about 3,50,000 mosques, and there are about 1.7 million Islamic religious leaders in the country who are available for the nearby population at least five times a day during the prayer times (Arafat 2024a). In a Muslim‐majority country, an acceptable attitude toward mental illness among Imams is supposed to increase the referral to mental health services and reduce the treatment gap in the community. However, no attempt has been noted to assess the attitude, social stigma, and literacy of mental health among Islamic religious leaders in Bangladesh. Therefore, we aimed to see attitudes, literacy, and social stigma toward mental illness among Islamic religious leaders in the country. It may identify the potential areas for further attempts to include traditional healers in the available mental health service providers at the primary care level of Bangladesh.

## 2 Methods

### 2.1 Data Collection

This was a cross‐sectional study. Data were collected from Sunni Imams working in different parts of Bangladesh from September 2024 to January 2025. We purposely selected Imams undergoing a training program arranged by the *Islamic Foundation, Bangladesh*. It arranges Regular Imam Basic Training with 45‐day duration for Islamic religious leaders (Imams, Muazzins, and Hafizs‐Qaris) working in a mosque (Arafat 2024a). Data were collected from several cohorts of the training.

### 2.2 Tools of Data Collection

#### 2.2.1 Semi‐Structured Questionnaire for Demographic Variables

This section has been adapted from a previous study conducted among the same population (Arafat, Islam, et al. 2024). It documented age (completed years), marital and academic status, family type, and income of the family in a month, family history of psychiatric disorder and consumption of psychiatric medications, personal history of psychiatric disorders, and personal consumption of psychiatric medications.

#### 2.2.2 Self‐Reporting Tool Assessing Attitude and Social Stigma

We adapted this section in Bangla from Hussain's (2022) study conducted in the USA among Shia Muslims (Hussain 2022). The original instrument has 24 items under eleven sections (biological cause—3 items, sinful cause—3 items, spiritual influence—2 items, higher power [Allah] influence—2 items, external causes—3 items [Jinn, evil eye, and black magic], individual reasons—4 items [environment, family, discrimination stress, and religious treatment], and social stigma‐7 items). In the scale, 20 items were selected from the Community Attitudes Toward Mentally Ill (CAMI) scale, three items were created by the author, and one item was selected from the American Psychological Association (APA) survey. We dropped two items (alcohol and discrimination stress/religious persecution) as alcohol is not widely used like USA and more than 90% of the people are Muslims in Bangladesh. The instrument assessed risk factors for psychiatric disorders in several areas and social stigma toward persons with mental illness. Therefore, this section contained 22 items under ten sections (biological cause—3 items, sinful cause—2 items, spiritual influence—2 items, higher power [Allah] influence—2 items, external causes—3 items, individual reasons—3 items [environment, family, and religious treatment], and social stigma‐7 items). Item‐wise responses were noted in 5‐point Likert Scale categorized as *strongly disagree*, *disagree*, *neutral*, *agree*, and *strongly agree*.

#### 2.2.3 Self‐Reporting Tool Assessing Literacy of Mental Illness

We adapted this section in Bangla from Hussain's (2022) study conducted in the USA among Shia Muslims (Hussain 2022). The original instrument contains four items. However, we dropped one item (nutrition) and added two items (anxiety disorder and obsessive–compulsive disorder [OCD]), making the questionnaire five items that is, depression, anxiety, OCD, schizophrenia, and self‐harm/suicidal behavior. Item‐wise responses were noted in three points (*yes, no, don't know*).

### 2.3 Statistical Analysis

Statistical analyses were performed using Statistical Package for the Social Sciences version 25. Frequencies and percentages were used to sum up sociodemographic factors and each item in the survey. The Shapiro–Wilk test was conducted to test the normality of the data. The data were found to be non‐normally distributed, and so the median and interquartile range (IQR) for each of the subscales were calculated. To determine bivariate associations between sociodemographic characteristics and causes of mental illness, we performed the Mann–Whitney test and the Kruskal–Wallis test for dichotomous variables and polychotomous variables, respectively.

### 2.4 Ethical Considerations

We collected ethical approval for the study from the ethical review committee of Enam Medical College in July 2023 (EMC/ERC/2023/07‐1). We collected data after the participants had signed the informed consent form.

## 3 Results

### 3.1 Sociodemographic Characteristics

We collected data from 358 Imams with a mean (±SD) age of 31.7 (±9.9) years. The age of the participants ranged from 20 to 65 years. The majority of the participants were aged 30 years or younger (54.5%), were married (68.4%), maulanas (59.8%), and lived in joint families (57.3%). Among the respondents, 8.1% had a history of psychiatric illness, 5% had taken medications for mental illness, 17.3% had a family history of psychiatric disorder, and 16.8% had a family history of consuming medications for mental illness (Table 1).

**TABLE 1:** Sociodemographic characteristics of the participants (*n* = 358).

Variable	Category	*N*	%
Age (in years)	≤ 30	195	54.5
	31–49	113	31.6
	≥ 50	21	5.9
	Missing data	29	8.1
Marital status	Married	245	68.4
	Unmarried	105	29.3
	Divorced/separated	5	1.4
	Missing data	3	0.8
Level of education	Hafez	71	19.8
	Maulana	214	59.8
	Mufti	64	17.9
	Missing data	9	2.5
Family type	Nuclear	138	38.5
	Joint	205	57.3
	Missing data	15	4.2
Monthly family income (in BDT)	< 10,000	75	20.9
	10,000–29,999	176	49.2
	≥ 30,000	41	11.5
	Missing data	66	18.4
Personal history of mental illness	Present	29	8.1
	Absent	325	90.8
	Missing data	4	1.1
Life time history of taking psychotropic	Yes	18	5
	No	336	93.9
	Missing data	4	1.1
Family history of mental illness	Present	62	17.3
	Absent	289	80.7
	Missing data	7	2
Psychotropic taken by family members	Yes	60	16.8
	No	295	82.4
	Missing data	3	0.8

### 3.2 Attitude and Social Stigma

Mental illness is perceived as highly biological, influenced by sinful acts, has high spiritual influence, and is poorly associated with external issues like jinn, evil eye, and black magic by the participants (Table 2). About 87% of the participants agreed (agree + strongly agree) that mental illness is a disease, and about 75% agreed that mental illness is caused by a chemical imbalance of the brain. However, about 57% agreed that it is inherited, and more than 29% disagreed (disagree + strongly disagree) on this item (Table 2). More than 71% of Imams considered that sinful acts can cause mental illness, and about 89% thought that substance abuse can cause psychiatric disorders (Table 2). Strong faith was considered the best treatment by 72.4% Imams, and spirituality was considered preventive measure by 82.4% of participants, and 57.6% of the participants thought that Muslims should visit a religious leader before attending a psychiatrist. About 68% of the Imams thought that mental illness comes from Allah as a test, 55.1% disagreed with the causal role of jinn possession, and about 48% disagreed with the causal role of evil eye, while about 49% agreed on the causal role of black magic.

**TABLE 2:** Distribution of responses for attitude and social stigma toward mental illness among religious leaders of Bangladesh.

Items	Strongly disagree	Disagree	Neutral	Agree	Strongly agree	Missing data
Biological perspectives	*n* (%)	*n* (%)	*n* (%)	*n* (%)	*n* (%)	*n* (%)
Mental illness is a disease	23 (6.4)	11 (3.1)	11 (3.1)	176 (49.2)	135 (37.7)	2 (0.6)
Mental illness is usually inherited	20 (5.6)	84 (23.5)	46 (12.8)	170 (47.5)	33 (9.2)	5 (1.4)
Mental illness is caused by a chemical imbalance in the brain	15 (4.2)	29 (8.1)	28 (7.8)	205 (57.3)	63 (17.6)	18 (5.0)
Sinful perspective						
Sinful actions are often the cause of mental illness	24 (6.7)	46 (12.8)	27 (7.5)	189 (52.8)	66 (18.4)	6 (1.7)
Substance use can lead to mental illness	15 (4.2)	12 (3.4)	5 (1.4)	191 (53.4)	127 (35.5)	8 (2.2)
Spiritual perspective						
The best treatment for mental illness is strong faith	16 (4.5)	37 (10.3)	33 (9.2)	162 (45.3)	97 (27.1)	13 (3.6)
Being close to Allah prevents mental illness	14 (3.9)	18 (5.0)	26 (7.3)	170 (47.5)	125 (34.9)	5 (1.4)
Muslims should go to a religious leader with mental health issues before a mental health professional	46 (12.8)	52 (14.5)	48 (13.4)	137 (38.3)	69 (19.3)	6 (1.7)
Higher power perspective						
If a person becomes mentally ill, it is often the will of Allah	24 (6.7)	38 (10.6)	41 (11.5)	166 (46.4)	77 (21.5)	12 (3.4)
Mental illness is a test from Allah	17 (4.7)	47 (13.1)	39 (10.9)	168 (46.9)	76 (21.2)	11 (3.1)
External perspective						
Mental illness is often a result of possession by a jinn	55 (15.4)	142 (39.7)	60 (16.8)	69 (19.3)	18 (5.0)	14 (3.9)
Mental illness can be caused by the evil eye	48 (13.4)	123 (34.4)	54 (15.1)	97 (27.1)	19 (5.3)	17 (4.7)
Mental illness can be caused by the use of black magic	42 (11.7)	87 (24.3)	44 (12.3)	135 (37.7)	40 (11.2)	10 (2.8)
Social stigma						
Those with mental illness can get better with treatment	16 (4.5)	17 (4.7)	15 (4.2)	166 (46.4)	138 (38.5)	6 (1.7)
The mentally ill should be isolated from the rest of the community	146 (40.8)	115 (32.1)	25 (7.0)	45 (12.6)	20 (5.6)	7 (2.0)
The mentally ill are a burden on society	127 (35.5)	111 (31.0)	36 (10.1)	43 (12.0)	24 (6.7)	17 (4.7)
The mentally ill are less of a danger than most people think	80 (22.3)	101 (28.2)	72 (20.1)	69 (19.3)	21 (5.9)	15 (4.2)
Less emphasis should be placed on protecting the public from the mentally ill	92 (25.7)	111 (31.0)	60 (16.8)	64 (17.9)	20 (5.6)	11 (3.1)
It is best to avoid people with a mental illness	113 (31.6)	119 (33.2)	34 (9.5)	50 (14.0)	24 (6.7)	18 (5.0)
Individual questions						
If the environment is bad, anyone can become mentally ill	39 (10.9)	38 (10.6)	24 (6.7)	176 (49.2)	73 (20.4)	8 (2.2)
One of the main causes of mental illness is a lack of self‐discipline and willpower	34 (9.5)	46 (12.8)	48 (13.4)	159 (44.4)	66 (18.4)	5 (1.4)
Mental illness usually comes from tensions in the family	36 (10.1)	36 (10.1)	36 (10.1)	175 (48.9)	70 (19.6)	5 (1.4)

A favorable scenario was noted while assessing the social stigma of mental illness, while persons with mental illness were considered as dangerous (Table 2). About 85% of participants thought that mental illness improves with treatment, about 73% disagreed with the social isolation of persons with mental illness, 66.5% disagreed with the burdensomeness of persons with mental illness, and about 65% disagreed with the avoidance of mixing with persons with mental illness (Table 2).

About 63% of participants considered a lack of personal discipline, about 70% attributed to a bad environment, and 68.5% considered family tension as a causal factor for mental illness (Table 2). No significant association was noted between sociodemographic variables and attitude, and social stigma assessed by the Mann–Whitney test and Kruskal–Wallis test.

We found extremely favorable MHL status among Imams regarding depression, anxiety, OCD, schizophrenia, and suicidal behavior (Table 3). About 90% of the participants identified anxiety symptoms, 92.5% identified depression, 83% identified OCD, 88% identified schizophrenia, and 95% thought that persons with suicidal behavior should seek mental health support (Table 3).

**TABLE 3:** Mental health literacy status among religious leaders in Bangladesh.

Disorders	Yes	No	Do not know	Missing data
	*n* (%)	*n* (%)	*n* (%)	*n* (%)
Feeling sad, loss of pleasure in activities, loss of appetite, and sleep disturbances are symptoms of depression	331 (92.5)	9 (2.5)	17 (4.7)	1 (0.3)
Too much thinking, worrying excessively, and feeling nervous are symptoms of anxiety	321 (89.7)	24 (6.7)	10 (2.8)	3 (0.8)
Having the same unwanted thoughts repeatedly and doing the same thing over and again are symptoms of obsessive–compulsive disorder	297 (83.0)	24 (6.7)	34 (9.5)	3 (0.8)
A person with schizophrenia may see and hear things that nobody else sees and hears	315 (88.0)	26 (7.3)	15 (4.2)	2 (0.6)
If a person is threatening to harm themselves or others, they should be taken to a mental health professional	340 (95.0)	7 (2.0)	9 (2.5)	2 (0.6)

## 4 Discussion

### 4.1 The Major Findings of the Study

The study revealed a mixed attitude and social stigma toward mental illness and favorable MHL among Islamic religious leaders in Bangladesh. A favorable scenario was noted while assessing the social stigma of mental illness, except that persons with mental illness were considered dangerous. It identified a favorable MHL status among Imams regarding depression, anxiety, OCD, schizophrenia, and suicidal behavior. No significant association was noted between sociodemographic variables and attitude, and social stigma.

Biological and spiritual factors were perceived as important, whereas jinn possession and evil eye impacts were considered less important, which is different from another study conducted in Australia among Arabic and Christian religious leaders, where psychosocial aspects were noted as prominent factors (Youssef and Deane 2013). The high approval of spiritual factors could be explained by Islamic faiths, where it is considered that everything happens as per the wish of Allah (Ciftci et al. 2013). One multicountry study noted that lower stigma and higher religiosity were significantly related to a more favorable help‐seeking pattern (Fekih‐Romdhane et al. 2023). Another study identified spirituality as one of important factors for mental help‐seeking behavior as per the theory of planned behavior (Badran et al. 2024). The distribution of responses was also different from Hussain's (2022) study conducted among Shia Muslims in the USA. This may be due to the variations of participants, their educational backgrounds, and the measuring instruments.

External factors like jinn possession, black magic, and evil eye are pertinent in the Bangladeshi cultural context. Mullick et al. (2013) found that 61%, 50%, and 44% of patients believed that jinn possession, black magic, and evil eye, respectively can cause mental illness, which is different from this study. Here, Imams disapproved of the role of jinn possession and evil eye; however, they still considered the role of black magic in causing mental illness. A similar lower approval was also found in a study in the USA among Shia Imams (Hussain 2022). This finding is unexpectedly lower based on the sociocultural and educational background of the Imams. There may be possibilities of social desirability bias, while responding to the items of external forces. Additional qualitative studies would be beneficial to explore the perception. A similar perception was noted in an Australian study (Youssef and Deane 2013). These complex scenarios can be explained by the presence of discussions about *jinn*, *shaytaan*, demons, *bhut* (evil spirits), and *farista* (angels) in Islamic texts (Ghuloum et al. 2024; Dein and Illaiee 2013; Tanhan and Young 2022; Ibrahim and Whitley 2021).

Although MHL is a newer entity in Bangladesh, this study revealed an excellent status in identifying depression, anxiety, OCD, schizophrenia, and management perspectives of suicidal behavior (Arafat, Giasuddin, et al. 2024, Arafat et al. 2018). A similar favorable status was also noted in a study in the USA among Shia Imams (Hussain 2022). This may be due to the pattern of questions and their response options. Further studies are warranted to understand the dynamics.

### 4.2 Implications of the Study

The findings of this study have several implications. First of all, there is an extreme dearth of studies assessing the attitude and social stigma among Islamic religious leaders across the world. In that case, this study adds one of the few perspectives from a Muslim‐majority country, Bangladesh, the fourth leading country in terms of the number of Muslims and mosques. Subsequently, it indicates a favorable approach to the medical model of mental illness, the role of spirituality on mental health, along with disagreement with jinn possession and evil eye as prominent causative factors. This demonstrates the feasibility of initiating and maintaining formal collaboration with enduring mental health services. This could be an immediate action as a good proportion of patients visit religious and traditional healers before reaching mental health professionals (Giasuddin et al. 2012; Arafat 2024b; Meran and Mason 2019; Long and Ansari 2018; Ali and Milstein 2012). It is expected to increase the referral to the existing mental health facilities as there is no functional referral system in the country (Arafat 2024b; Wang et al. 2003). Additionally, Imams have had low stigma toward persons with mental illness, which is promising to engage them as mental health advocates, which will help to reduce stigma among the general population (Ayvaci 2016).

### 4.3 Limitation

To the best of the authors’ understanding, this is the first study assessing attitude, social stigma, and literacy among Imams in Bangladesh. However, the study has several limitations. First, this study was conducted using a cross‐sectional design that limits causal association among the variables. Second, data were collected by self‐reporting measures that may raise concerns of social desirability biases. Third, the tool was not culturally validated, which may be a source of biases in measuring the attitude, social stigma, and literacy. Fourth, we collected data by purposive sampling, which may raise concerns about the generalization of study results. Fifth, participants were only male in this study, which may create concerns about attitudinal variation based on gender.

## 5 Conclusions

This study revealed findings related to attitude, social stigma, and literacy among religious leaders in a Muslim‐majority country. Studies assessing the association between these variables and referral patterns to mental health services are warranted to consider the findings in policymaking. At the same time, the readiness and availability of mental health service providers at the primary and secondary care levels should be ensured.

## Author Contributions

S.M.Y.A. conceived the study. S.M.Y.A., M.K.I. designed and performed the study. M.K.I., B.M., M.G.K. analyzed the study data. All authors contributed to the interpretation of results. S.M.Y.A., M.G.K., S.T.K, drafted the manuscript, while M.K.I. and B.M. reviewed and edited the draft. All authors read and approved the final manuscript and agreed to be accountable for all aspects of the work.

## Disclosure

The lead author, S. M. Yasir Arafat, affirms that this manuscript is an honest, accurate, and transparent account of the study being reported; that no important aspects of the study have been omitted; and that any discrepancies from the study as planned have been explained.

## Conflicts of Interest

The authors declare no conflicts of interest.

## Peer Review

The peer review history for this article is available at https://publons.com/publon/10.1002/brb3.70998

## Data Availability

Data will be provided to the query request of the corresponding author.
